# Patient and public involvement in basic and clinical psychiatric research: a scoping review of reviews

**DOI:** 10.1186/s12888-025-06608-7

**Published:** 2025-03-25

**Authors:** Anne Werner, Julia Lauberger, Anke Steckelberg, Gabriele Meyer

**Affiliations:** Institute of Health and Nursing Science, Medical Faculty of Martin Luther University Halle- Wittenberg, University Medicine Halle, Magdeburger Straße 8, 06112 Halle (Saale), Germany

**Keywords:** Patient participation, Psychiatry, Psychology

## Abstract

**Background:**

Patient and public involvement (PPI) in health research has become established as an essential component of international health research. Particularly, patients and stakeholders’ commitment to psychiatric research faces various challenges. This scoping review aimed to examine the existing literature to identify the aims, methods, barriers, and facilitators of PPI in clinical and basic psychiatric research.

**Methods:**

This scoping review’s methods were guided by the framework proposed by Arksey and O’Malley. The literature search was conducted between October and November 2023 on six databases (MEDLINE, Web of Science, EMBASE, PsycInfo, PubPsych, and the Cochrane Library). We included reviews that summarized the results of primary studies describing methods for PPI in psychiatric scientific research, regardless of their subjects’ underlying psychiatric conditions and the primary research context (clinical or preclinical). The inclusion criteria included a description of the methods, components, and characteristics of participation in psychiatric research. We included reviews published between 2008 and 2023 regardless of participants’ language, country, or age.

**Findings:**

Twenty reviews comprising 429 studies were included. They revealed that PPI was used to pursue various objectives (e.g., prioritizing research questions). Common methods included focus groups, advisory boards, workshops and interviews. Only one review reported financial compensation for those involved. PPI ranged from tokenism to involvement in data analysis and the dissemination of findings. Facilitators and barriers were identified in relationship and communication factors, organizational and practical factors, and in (co-) researchers training. The most frequently mentioned facilitators of successful PPI were trust and strong relationships. The most frequently mentioned barrier was the power imbalance between the participants and researchers. We identified positive and potential negative effects of PPI.

**Conclusion:**

Golden rules for practice (clinical and basic research) derived from the results are as follows: (I) Foster a culture of collaboration and mutual respect between researchers and PPI participants. (II) Provide adequate resources and support for PPI activities, including funding and training programs. (III) Develop clear guidelines and standards for PPI to ensure consistency and quality. (IV) Develop a willingness to integrate PPI into all phases of research, from planning to the dissemination of results.

**Scoping review registration:**

10.17605/OSF.IO/7YS5C.

**Supplementary Information:**

The online version contains supplementary material available at 10.1186/s12888-025-06608-7.

## Background

Patient and public involvement (PPI) in clinical research has become an internationally established, essential component of health research [1–3]. PPI is often defined as research conducted ‘with’ or ‘by’ patients and the public, rather than ‘about’ them [4–6]. PPI can involve different levels of participation, from mere consultation to genuine involvement (e.g. decision-making competencies in sub-areas of research), and can extend to different stages of the research process, from the identification of research topics to the publication and dissemination of results [4, 7, 8]. PPI is expected to improve the quality and relevance of research by ensuring that it is responsive to the public’s needs and concerns. PPI promotes democratic principles such as citizen participation, accountability, and transparency, and empowers those affected by research to help shape it. By involving the public and various stakeholders with different perspectives, research topics and methods become more practical and relevant [9].

Despite the evidence of PPI’s benefits, there are clear challenges associated with PPI [10] due to its complexity [11]. PPI (also named ‘community-based participatory research’ and ‘[youth] participatory action research’) can be carried out using different approaches. Particularly, patients and stakeholders’ engagement in psychiatric research presents specific challenges [4]. One of the main problems regarding patients with psychiatric illnesses is the power imbalance among researchers, psychiatrists, and other professionals. Furthermore, patients’ participation in research is often met with stigma—e.g., the preconception that people with psychiatric illnesses are cognitively impaired and incapable of producing knowledge [4, 12]. This can lead to a gap between the requirements of research and the perspective of those affected [4, 12]. This gap can be reinforced by problems in PPI’s financing. For example, insufficient financing can aggravate power imbalances [4]. The reduction of co-researchers to a supply of knowledge from a specific field of experience also represents a challenge for psychiatric patients’ participation [4].

PPI in German psychiatric research represents a relatively nascent area of research improvement [4, 7]. Consequently, it seems prudent to aggregate the methods specifically employed in this domain with PPI’s overarching goal while identifying potential facilitators and barriers.

A scoping review was conducted to support the constitution of the German Centre for Mental Health (*Deutsches Zentrum für Psychische Gesundheit* [DZPG]). The DZPG aims to improve and democratize mental health research and care in Germany by engaging patients and their families through PPI. A trialogue center council consisting of patients, relatives, and researchers was established in 2021. This involvement was intended to improve the quality, relevance, and credibility of the DZPG’s research. The central premise is that PPI is expected to be embedded in all phases of research, from research planning to the dissemination of results. The DZPG plans to establish a PPI department that will serve as supporting infrastructure. Additionally, people engaged in PPI and their families will be involved in calls for proposals, reviews, and selection procedures. New appointments to scientific positions will also consider applicants’ experience and expertise. The aim is to establish PPI nationally and make it visible and networked internationally. The results of this scoping review will contribute to the development of PPI and the accompanying processes within the DZPG [7, 13].

Our scoping review was embedded in the establishment of a PPI structure within the DZPG [14]. The DZPG has several locations in Germany and is funded by the Federal Ministry of Education and Research. The DZPG aims to pool scientific and practical expertise to create multidisciplinary structures to improve care for people with mental illnesses [15].

This scoping review’s main objective is to provide an overview of PPI in basic and clinical psychiatric research. We consider a scoping review to be the appropriate methodology because it allows us to obtain a comprehensive overview regardless of the validity of individual reviews [2]. By drawing insights from the research findings, we aimed to synthesize the existing body of knowledge. Furthermore, we derive future research priorities from these findings and identify gaps in the existing literature.

## Methods

This review was conducted in accordance with the Joanna Briggs Institute’s guidelines [16]. This methodology is based on the framework of Arksey and O’Malley [8]. Additionally, this review followed the PRISMA Extensions for Scoping Reviews [17]. The review protocol was registered with the Open Science Framework on October 29, 2023 [18].

The patients and public involved are referred to below as co-researchers. This term includes all non-academic participants in the reviews (e.g., patients, relatives, and community members). Because the terms used for these individuals differed between the original reviews, we preferred to use a common term.

### Eligibility criteria

We included reviews that summarized the results of primary studies describing methods for the involvement of patients, their relatives, and patient representatives in psychiatric scientific research, regardless of patients’ underlying psychiatric conditions and the primary research context (clinical or preclinical research). A prerequisite for inclusion was an extensive description of the key methods, components, and characteristics of psychiatric research participation. Reviews were included regardless of participants’ language, country, or age. We excluded reviews published before 2008 because we expected limitations in the transferability of older concepts of PPI to the current context. Additionally, a 2009 report detailing an National Health Service (NHS) literature search from 2007 to 2008 did not include psychiatric PPI reviews [11].

### Search strategy and information sources

Our search terms were based on the ICD10 system for mental and behavioral disorders (F00–F99) and frequent psychiatric disorders [19, 20]. The search terms for basic psychiatric research were taken from the general basic research terms of Carroll et al. [21] and the homepages of various universities in Germany. The search strategy was developed in collaboration with AW and JL (both female) and discussed with members of a DZPG PPI-dialogue group consisting of patient representatives and advocacy representatives based in Jena/Halle/Magdeburg. This contributed to sharpening the search strategy and adding additional terms to our strategy. All search terms were discussed with AS and GM. Table 1 presents the PubMed search strategy. All other search strategies are shown in Supplementary File 1. We used the following databases for the systematic search: MEDLINE (PubMed), Web of Science Core Collection, EMBASE (Ovid), PsycInfo (Ovid), PubPsych and the Cochrane Library. The search (following planning and developing the search strategy) was conducted between October 27 and November 6, 2023. Moreover, we recorded one paper by DZPG authors, published in January 2024 [22].

**Table 1:** Search strategy (PubMed)

tiab: (A1 OR A2) AND (((B1 OR D1) AND C2) OR B2 OR C1) 2008–2023	
A1	(“patient involvement”) OR (“Patient & Public Involvement”) OR (“Patient and Public Involvement”) OR (“Public and patient partnership”) OR (“Public and patient involvement”) OR (“peer research”)
A2	(“community-based research”) OR (“participatory research”) OR empowerment OR (“capacity-building”) OR (“collaborative research”)
B1	psychiatr*
B2	(anxiety) OR (phobia) OR (phobic) OR (“post-traumatic stress disorder”) OR (PTSD) OR (eating disorder) OR (anorexia) OR (bulimia) OR (depression) OR (depress*) OR (“obsessive compulsive disorder”) OR (OCD) OR (psychosis) OR (bipolar) OR (mania) OR (schizophrenia) OR (adhd) OR (“attention deficit hyperactivity disorder”) OR (“substance abuse”) OR (addict*) OR (dementia) OR (“Alzheimer disease”)
C1	(drug development/exp OR (drug NEXT/1 development*)) OR (chemical analysis/exp OR (chemistry NEXT/1 techni*)) OR (gene* NEXT/1 research) OR (neuroanatom*) OR (behavioral pharmacolog*) OR (chemistr* NEXT/1 (neuro* OR bio*)) OR (metaboli* patholog*) OR (endocrinolog*) OR (MRI OR (“magnetic resonance imaging”)) OR (MRS OR (“magnetic resonance spectroscop*”)) OR (biomedicine/exp OR (biomedical NEXT/1 science*)) OR (simulation NEXT/1 (acustic OR visual)) OR (biomedicine/exp OR (biomedical NEXT/1 science*))
C2	(“clinical research”) OR (“preclinical research”) OR (“basic research”) OR (“basic scientific research”) OR (“laboratory research”)
D1	(“mental health”)

### Article selection

**Fig. 1:**
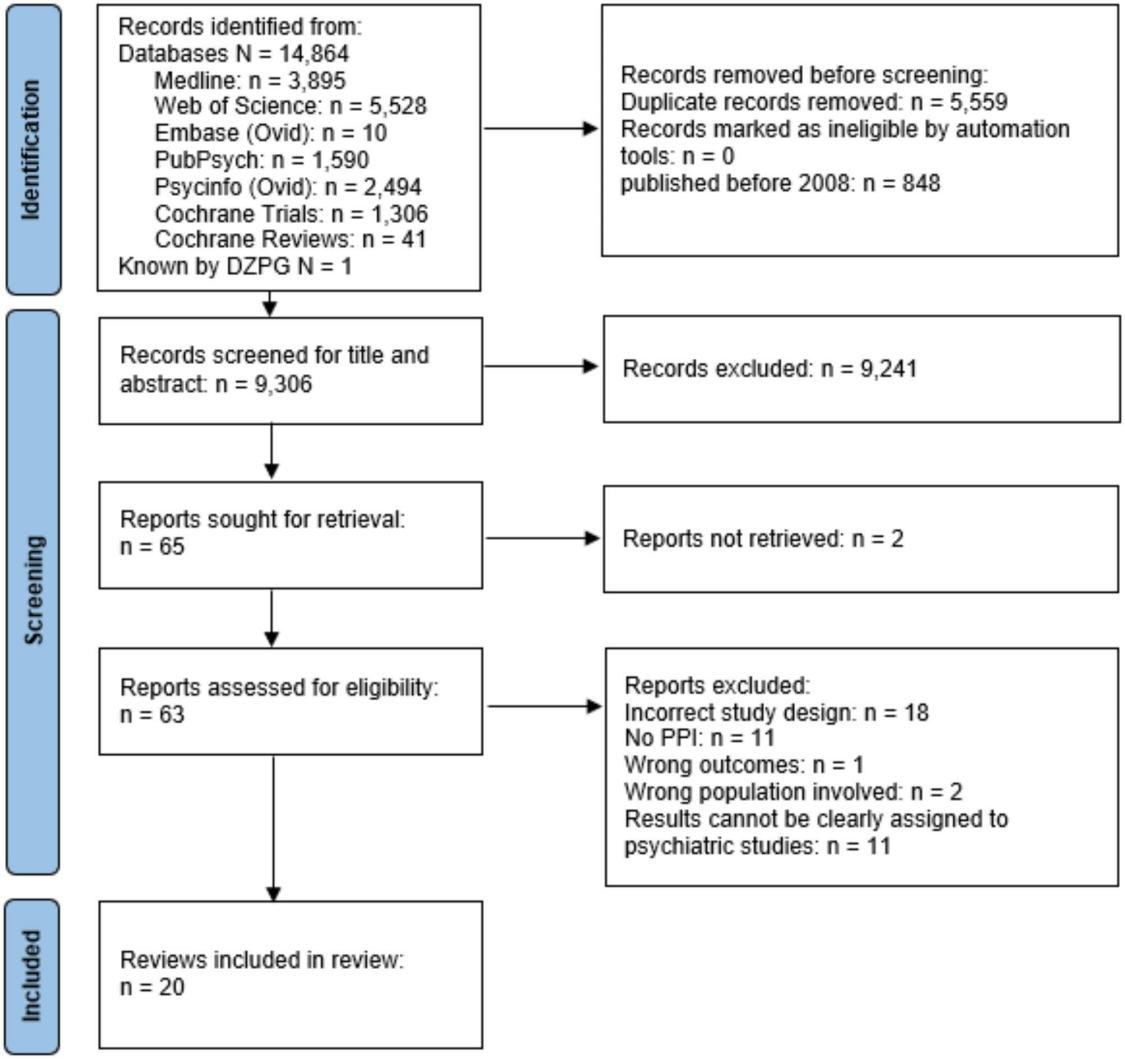
Flow chart of the identification relevant articles

All articles identified in the database search were uploaded to the research collaboration platform for literature and systematic reviews Rayyan [23]. All duplicates were removed by AW, JL, and two research assistants (female and male). Title and abstract screening were performed by JL, AW, and a research assistant (female). The reviewers independently screened the titles and abstracts. To verify that the inclusion and exclusion criteria were appropriate and applicable, the reviewers discussed their decisions after reviewing the 20 records. After a blinded review of the hits, discrepancies regarding inclusion and exclusion were resolved through discussions between AW, JL, and the assistant. If a consensus could not be reached among the reviewers, GM and AS were consulted for a resolution. Full-text screening was performed using the same procedure. First, blinded screening of the full text was performed by AW and JL. This was followed by a meeting to resolve any discrepancies; in case of conflict, GM and AS were consulted for advice. A flowchart for the identification of relevant articles is shown in Fig. 1.

### Data extraction and synthesis

The data extraction procedure was based on the findings of Carroll et al. and Maccarthy et al. and the PRISMA-ScR checklist [17, 21, 24]. A reviewer (AW) and a research assistant extracted the data. Different reviewers checked the extracted data for correctness. A quality assessment was not performed during data extraction. For each included study, we extracted the following information from an Excel spreadsheet: (1) study characteristics (country, sample size, language, date, and design); (2) characteristics of the included reviews (design, countries of included studies, timeframe, participant characteristics, and researchers’ characteristics); (3) scientific discipline/context (primary disease area and primary research area); (4) preconditions for PPI (rationale, aims, and preconditions); (5) methods/methodology (PPI implementation, format, and max. stage of PPI), (6) conditions (facilitators and barriers), and (7) evaluation (design, results, benefits, challenges, and PPI’s impact). To summarize these data, the extracted information for every outcome was copied into a word document and sorted by AW. After the first content-based classification and summary of facilitators and barriers, JL summarized the key points.

### Findings

We included 20 reviews with 429 individual studies, of which 65 were discussed in more than one review. The authors decided to include an additional review that emerged from the working group, which met the inclusion criteria but was not published until 2024, after the literature search was completed. Because of said study’s high level of relevance, we decided to include it. Said study covers another aspect of research on affected young people and comes from the DZPG context in which the results of this scoping review will also be used [22]. The 20 reviews analyzed were spread over a period of 15 years, with most being published in 2023 (five reviews) and 2017 (three reviews) (see Table 2). The primary target populations for the included reviews were patients with dementia/Alzheimer’s disease and general mental health issues (eight reviews each). Other areas included substance misuse (four), depression (two), psychosis (one), older people with learning disabilities (one), intellectual disability (one), domestic violence (one), quality of life (one), autism (one), and others (two). Most included reviews were scoping reviews (10), followed by systematic reviews (five), general reviews (three), and evidence syntheses (one). Based on these reviews, we identified, synthesized, and processed information on the preconditions and aims, methods of inclusion, and evaluation of PPI with their respective facilitators and barriers (Supplementary Files 2–4).

**Table 2:** Review characteristics

Category	Subcategory	*n*	Reference
Total		20	
Year of publication	2009	1	[25]
	2010	1	[26]
	2016	1	[27]
	2017	3	[28–30]
	2018	2	[31, 32]
	2019	2	[33, 34]
	2020	2	[35, 36]
	2022	2	[21, 37]
	2023	5	[38–42]
	2024	1	[22]
Primary disease area	Dementia/Alzheimer	8	[28, 30, 31, 33–35, 37, 41]
	Mental health & psychiatry in general	8	[21, 22, 26, 28, 31, 38, 39, 42]
	Substance use	4	[27, 36, 39, 42]
	Depression	2	[25, 31]
	Psychosis	1	[40]
	Older people with learning disabilities	1	[28]
	Intellectual	1	[29]
	Domestic violence	1	[32]
	Quality of life	1	[35]
	Autism	1	[21]
	Other	2	[30, 35]
Primary research area	Basic research	1	[21]
	Health service research	19	[22, 25–42]
Study design paper*	Scoping review	10	[21, 27, 33–35, 38–42]
	Systematic review	6	[22, 29, 31, 32, 36, 37]
	Literature review	3	[25, 26, 30]
	Synthesis of the evidence	1	[28]
Outcomes	Preconditions	9	( [21, 26, 29–31, 34–36, 41])
	Methods/ methodology	18	[21, 22, 25–27, 29–38, 40–42]
	Evaluation	18	[21, 22, 25–42]

* Self-reported study design

### Preconditions and aims

Nine reviews reported on the objectives of PPI use [21, 26, 29–31, 34–36, 41]. Carroll et al. reported PPI use following a funder directive [21]. Miah et al. and Valdez et al. analyzed PPI use to improve research objectives and prioritize research questions [34, 36]. Crocker et al. and Schilling and Gerhardus conducted reviews of studies on people living with dementia and reported PPI use to improve research methods (improve recruitment and the validity of results, interviews, and data) [30, 31]. Four reviews reported PPI use to ensure studies’ appropriateness and acceptability [21, 29–31]. Cowdell et al. reported PPI use to select content and language and address the limitations of previous interventions [35]. PPI has been used to understand (minority) communities’ lived experiences and needs, as highlighted by Reyes et al. and Stacciarini et al. [26, 41]. Reyes et al. and Schilling and Gerhardus reported PPI use to demonstrate the feasibility of co-researching with people with dementia [30, 41]. Schilling and Gerhardus and Carroll et al. reported PPI use to enhance resource utilization and the integration of different knowledge sources [21, 30]. Also mentioned were improvements to the target population [30], recognizing community members as important partners in mental health research [26], building youth capacity, reducing or delaying the onset of substance abuse in youth [36], and advising on the study results [29].

### PPI methods

#### Format

Fourteen reviews included information on PPI methods [21, 22, 25–27, 29–31, 33–35, 38, 40, 41]. Table 3 summarizes the different participation methods used.

**Table 3:** PPI format and reference

Format	Reference
Focus groups	[21, 22, 25, 26, 29–31, 34, 35, 38]
Steering committee/ feedback group/ advisory committee/reference group	[21, 22, 30, 31, 33, 40, 41]
Interviews	[21, 26, 29, 34, 35]
Workshops	[21, 30, 34, 35, 38, 40]
Different types of meetings and drop-in sessions	[25, 34, 38]
Questionnaire, survey, or postal feedback	[26, 30, 34]
Modified Delphi process combined with consensus conference & anonymous reader consultation	[30, 34]
Face-to-face methods (domiciles, small local groups)	[22, 30]
Ad hoc involvement for flexible one-time consultations	[22, 30]
PPI Event	[34]
Interactive web tool	[35]
Stakeholders’ discussions	[26]
Training (for co-researchers and/or lead researchers	[27, 37]

Five reviews reported forms of participation that did not represent authentic participation [25, 26, 29, 35, 38]. For example, participants were observed [25, 35], tested the prototypes at home [35], kept a personal diary [29], or attended lectures at a university [38].

Seven reviews mentioned co-researchers’ involvement in the research process [22, 25, 27, 33, 34, 38, 40]. Co-researchers participated in generating the research design [21, 25, 34, 35], conducting interviews or interventions [27, 29, 33, 35, 38], or were active in data analysis [25, 33–36, 41] and the dissemination of findings [21, 25, 34–36, 41].

#### Financial compensation

Five reviews dealt with the financial compensation for co-researchers [27, 29, 34, 37, 42]. Four reviews noted that financial or other compensation was discussed [27, 29, 34, 42]. Only Kowe et al. reported financial compensation for co-researchers in three studies [37]. However, a budget for financial compensation should be included in research planning [29].

#### PPI implementation and stage of involvement

Thirteen reviews reported the types of PPI implementation [21, 22, 25–27, 29–32, 34, 36, 37, 41]. These reviews identified different approaches to participation: PPI [21, 30, 34], community-based participatory research [22, 25–27, 32, 36], youth participatory action research [22, 36] and participatory action research [22, 29]. Crocker et al. mentioned bimonthly advisory board meetings [31], whereas Di Lorito et al. reported consultations every three months [29]. The use of frameworks and intervention models was noted by Carroll et al. and Valdez et al., but without specifications [21, 36]. Reyes et al. reported the use of two different governance models: a consensus governance model that included equal voting rights for researchers and co-researchers in decision-making, and a decentralized decision-making framework in which members represented different stakeholders in subcommittees responsible for specific aspects of the research process [41]. Valdez et al. mentioned the use of informal or formal agreements for shared decision making in several of the included studies (*n* = 8) [36].

Fourteen reviews reported the stage of co-researcher involvement [21, 22, 29, 30, 32–38, 40, 41]. Three different classification systems were identified: Arnstein’s classification [37, 43], Roger Hart’s ladder of participation [36], Wright’s stage model of participation [22], and levels of involvement adapted from Sellars et al. [22, 44].

Four reviews reported that the patients and the public were research partners with equal or nearly equal rights as academic researchers [22, 29, 30, 36]. The participants included older adults, individuals with intellectual disabilities, and young adults. In nine reviews, the authors identified the involvement of co-researchers at the level of co-authoring papers and dissemination of the results [21, 22, 29, 30, 32–34, 36, 41]. Four of these reviews included studies on people living with dementia [30, 33, 34, 41], while two reviews focused on young people [22, 36].

### Evaluation

#### Evaluation method

Three reviews identified evaluation methods for PPI use [32, 34, 38]. One review identified the evaluation of youth participatory action research vis-à-vis substance abuse among youths involved in research [36]. Only Ragavan et al. found information on PPI evaluations in each of the included studies using self-created measures [32]. Miah et al. reported evaluation methods for three of the 20 studies, employing open-ended questions, online surveys, semi-structured interviews, and focus groups [34]. Florence et al. reported the use of Guba and Lincoln’s fourth-generation evaluation in seven studies [38, 45]. According to Miah et al., the quality of evidence of PPI’s impact and benefits is low [34].

#### Facilitators and barriers

Sixteen reviews reported facilitators of or barriers to PPI implementation [21, 22, 25, 27–30, 32–34, 36–40, 42] (Table 4).

**Table 4:** Overview of facilitators and barriers

Categories	Facilitators	Barriers
I. Relationship and communication factors	1. Strong relationships and trust	1. Skepticism of the academic researchers
	2. Defining clear roles	2. Power imbalances
II. Organizational and practical factors	1. Adaptation of materials and methods	1. Problems with recruitment
	2. Good planning	2. Insufficient financial planning
	3. Anticipatory budgeting	3. Practical challenges
	4. Adequate meeting strategies	4. Timing and scheduling challenges
	5. Collaborative time management	
III. (Co-) Researcher’s training	1. Training and content for academic researchers and co-researchers	1. Practical challenges and access to resources
		2. Financial constraints

#### Relationship and communication factors

##### Facilitators

Thirteen reviews reported on facilitators that can be subsumed under the category “human and communication factors” [21, 22, 25, 27–30, 32, 33, 36, 39, 40, 42].

*Strong relationships and trust*

A cornerstone of PPI’s success is the building of strong relationships and trust [21, 22, 25, 27, 29, 30, 32, 33, 36, 39, 42]. This can be achieved by creating an informal environment [22, 33, 40] and ensuring that researchers and co-researchers spend time outside of formal research settings [22, 29, 30], engaging in informal conversations [32]. Such interactions can help researchers understand co-researchers’ daily lives and provide the latter with a platform to express their feelings [29]. Safe, non-judgmental environments are especially beneficial for engaging with people with substance abuse issues, ensuring that they feel accepted and valued [27, 42]. Particularly, when working with people living with dementia, who may experience difficulties bonding with others, it is important to ensure the regular renewal of the relationship, as suggested by Schilling and Gerhardus [30]. Burton et al. mentioned that dementia-specific communication training is useful for scientists in this context [33]. Di Lorito et al. suggested that nonverbal communication is an important factor [28, 29]. Maintaining a calm demeanor and avoiding making assumptions or finishing peer researchers’ sentences are effective communication and relationship strategies [28, 30]. Formal recognition of contributions and feedback on how co-researchers have influenced the research can also be a vital component of the process, as reported by three reviews [33, 37, 42]. Di Lorito et al. recommended that emotional support and counselling be provided to those with intellectual disabilities [28, 29]. Keeping caregivers informed about the involvement of co-researchers helps maintain supportive networks [29]. Di Lorito et al. also recommended keeping the caregivers of co-researchers with intellectual disabilities informed about their involvement [29]. According to Jakobsson et al., it is important to explain how data will be stored, especially to co-researchers with recent psychotic episodes [40].

*Defining clear roles*

Defining clear roles and ensuring continuity are important for maintaining structured collaboration, as indicated by eight reviews [22, 25, 28–30, 33, 40, 42]. Di Lorito et al. and Totzeck et al. recommended discussing researchers and co-researchers’ roles, responsibilities, and mutual expectations [22, 28], as conflicting views are reported to be a frequent challenge in PPI [42]. Di Lorito et al. reported that roles changed over time because of the co-researchers’ increasing confidence in their abilities [29]. Additionally, Totzeck et al. recommended that when working with young people, their contributions should be dynamic and flexible [22].

##### Barriers

Despite these facilitators, several barriers can impede effective communication and relationship building in PPI. Eleven reviews reported on potential challenges related to relationships and trust [21, 22, 27–30, 33, 36, 38, 40, 42].

*Skepticism of the academic researchers*

Researchers are skeptical about involving co-scientists in preclinical research, a field in which long-term commitment by co-researchers is especially important, according to Carroll et al. [21], as they tend to value their academic knowledge more than their experiential knowledge, as reported by Sheikhan et al. and Florence et al. [38, 42].

*Power imbalances*

Five reviews reported that power imbalances between academic researchers and co-researchers could lead to conflict and hinder true collaboration [28, 30, 38, 40, 42]. Two reviews found that researchers’ disregard for co-researchers’ expertise can lead to tension and mistrust, thus disrupting the working relationship [38, 42]. Furthermore, co-researchers with dementia or cognitive impairment may struggle with memory problems and slow cognitive processing, thus leading to difficulties in fully engaging in research [28–30, 33]. This can lead to co-researchers with dementia tending to agree with the researchers [30]. When involving older people, their limited mobility can be a barrier to their participation [30]. Valdez et al. reported that researchers were not always willing to involve their young co-researchers in the early stages—e.g., in developing research questions or designs [36]. Moreover, researchers’ prejudices or factors on the side of co-researchers have been reported as potential barriers. Meanwhile, Jakobsson et al. and Totzeck et al. highlighted the importance of focusing on mental health stigma when working with adolescents or adults with psychosis [22, 40]. However, positive tests for illicit substances, homelessness, or previous arrests can be barriers to co-productive research involving people who use drugs [27]. It may be necessary to exclude people who test positive for illicit drugs or who are too intoxicated, as they pose a threat to researchers’ safety [27].

#### Organization and practical factors

##### Facilitators

Ten reviews identified several organizational and practical factors related to PPI as potential facilitators [22, 25, 27–30, 33, 39, 40, 42].

*Adaptation of materials and methods*

Different types of adaptations were listed in nine reviews [22, 28–30, 33, 35, 39, 40, 42], depending on co-researchers’ needs: For co-researchers with dementia, four reviews suggested visual adaptations such as the use of large fonts, black print on colored paper, word cards, storyboards, pictures, visual prompts and laminated cards [28, 30, 33, 35]. Totzeck et al. recommended the use of pictures for younger children [22]. Linguistic adaptations, including the use of simple language, avoiding jargon, reducing abstract language, and minimizing the use of complex concepts were identified as additional options [28, 30, 39, 42]. Making information accessible and consolidating knowledge through refresher sessions and summaries might also be beneficial [30]. The use of methods tailored to the target group, such as role-play and flexible communication channels, can potentially increase commitment [22, 33]. According to Totzeck et al., if younger people are involved, mutual respect and the feeling of involvement is an important factor [22].

*Good planning*

Totzeck et al. also stated that good planning is essential for successful PPI, and that the level of participation and a demand-driven study design should be planned before the project starts [22]. Therefore, the use of PPI guidelines is recommended [22]. If younger participants are involved, study information and informed consent should be provided in plain language to the parents [22]. Age, gender, and cultural aspects should be considered when recruiting adolescents for PPI [22], and the involvement of co-researchers should take place at an early stage of the research process [42]. Records should be maintained to analyze co-researchers’ influence on research development [22]. Collaborative research requires leadership commitment to PPI and institutional support [39].

*Anticipatory budgeting*

Five reviews recommended considering fair compensation for co-researchers’ time, travel [27, 29, 33, 37, 42], or salary [27, 29] during budget planning. Budgets should also consider the need for additional training sessions and team-building events [22]. One review pointed out that co-researchers may need assistance with travel arrangements or support to enable them to attend research meetings [29]. Late reimbursement of expenses further complicates involvement, particularly for co-researchers [33].

*Adequate meeting strategies*

Effective meeting strategies include early planning of team meetings, setting a meeting agenda, coordinating meetings, maintaining contact between meetings, and using pre-research and debriefing sessions, as stated in four reviews [29, 33, 39, 42]. Extra time and sensitive facilitation can help encourage contributions, especially from people with dementia or younger people [22, 33]. Meetings for young people should not be scheduled for weekends or school holidays [22]. As suggested by Totzeck et al., meetings should be evaluated to address misunderstandings [22]. The location of the meeting is also important, as seven reviews indicated: University environments can be challenging for certain groups, such as people with dementia or older adults [22, 25, 28–30, 33, 40]. Meetings can also be held in more familiar or accessible settings [28, 30, 33]. Outdoor spaces and clearly structured, quiet, and neutral locations were supportive [25, 30]. Youth-friendly locations and refreshments are recommended for meetings involving young people [22].

*Collaborative time management*

Another important factor is time management, which was reviewed in three studies [22, 30, 33]. Additional time should be allocated for the entire project and teambuilding activities [22]. Two reviews recommended allowing extra time for meetings so that co-researchers feel confident about participating, and more breaks could be allowed if necessary [30, 33]. Methods can be adapted to reduce the time required, such as postal consultations instead of face-to-face meetings [30]. For participants with a psychiatric diagnosis, it may be beneficial to schedule participation during remission rather than during the acute phase [22]. Practical work with co-researchers should be evaluated to optimize investments in time and resources [28].

##### Barriers

Twelve reviews identified several barriers to PPI related to organizational and practical factors [21, 22, 25, 28, 30, 32–34, 36, 39, 40, 42]. Burton et al. found that challenges are often reported on the part of researchers [33] but there are also barriers found on the side of the co-researches with mental health or substance abuse issues in their clinical history [30].

*Problems with recruitment*

Six reviews described difficulties in recruiting co-researchers [21, 28, 30, 33, 36, 42], and certain groups such as people with severe dementia and adolescents were reported to be particularly difficult to reach [33, 36]. Difficulties in recruitment can lead to a lack of diversity and representativeness among co-researchers, as indicated by six reviews [21, 28, 30, 33, 39, 42]. Cultural barriers were identified [22, 33], such as the exclusion of non-English speakers if funding for interpreters was lacking [33].

*Insufficient financial planning*

Financial challenges, such as delayed reimbursements and increased research costs for compensation and training, are also potential barriers within this context [28, 32, 33]. Funds for the interpreters were required to avoid participation bias [33]. General support from funding bodies and increased and more flexible funding for PPI is needed [39, 42].

*Practical challenges*

Practical challenges include the need for clear and early planning [39] and management of large PPI groups [33]. The competitive nature of research [42] and long-term commitment required for preclinical research can hinder PPI [21]. High turnover among mental health professionals and overworked staff can also impact the relationships and success of collaborative research [32, 40].

*Timing and scheduling challenges*

Reported barriers due to time-related aspects include busy schedules, tight research agendas (especially for people with dementia), childcare and finding a suitable time for all participants to attend [22, 25, 30, 32–34]. For older co-researchers, only engaging in short research periods may be feasible because they become exhausted more quickly [30].

#### (Co-) Researchers training

Seven reviews identified aspects of training that can serve as potential facilitators [22, 27, 29, 33, 37, 39, 42].

##### Facilitators

*Training and content for academic researchers and co-researchers*

Four reviews mentioned training for academic researchers to facilitate PPI’s successful implementation [22, 37, 39, 42]. Hawke et al. emphasized the need for training for researchers and research staff to avoid tokenism and encourage the real involvement of co-researchers with a history of substance abuse [39]. Five reviews addressed training for co-researchers as a helpful tool for implementing PPI and equipping participants with the necessary skills to participate in research [22, 27, 29, 37, 39]. It is recommended to train co-researchers in technical skills such as handling information sheets, consent forms and conducting interviews, as well as relational skills such as listening and interacting with people from different backgrounds [29].

One-on-one training sessions and workshops, as well as co-researchers–researchers pairings for initial and ongoing training can ensure continuous skill development [39]. Additionally, integrating young people into the design and delivery of training programs can enhance the relevance and effectiveness of these sessions [22]. Burton et al. reported one study that identified the individual skills of each group member to help them choose targeted tasks or provide support in areas they are unsure about [33, 46].

##### Barriers

*Practical challenges and access to resources*

Three reviews mentioned practical challenges that could hinder effective training for researchers and co-researchers [21, 33, 39]. For example, it is difficult to access training resources, provide appropriate training and mentors [21, 33, 39].

*Financial constraints*

The increased costs associated with providing training, compensating peer researchers, and supporting their participation can strain research budgets [28, 32].

#### PPI’s impact

Mostly anecdotal effects [34] have been reported; only a few studies with formal evaluation have reported on PPI’s effect, while evidence quality is low [34], and there are no quantitative data on how researchers experience PPI [22].

#### Impact on research

Twelve reviews described PPI’s positive impact on the research process [21, 26–29, 31–34, 37, 40, 42]. Three reviews reported PPI’s nonspecific positive impact [27, 32, 42]. Four reviews reported increasing recruitment or lower attrition rates when co-researcher feedback was implemented [28, 31, 33, 34, 42]. In contrast, one review reported no significantly higher enrollment rates for PPI interventions [31]. Five reviews reported on PPI’s impact on research outcomes or materials (e.g. make a website more accessible, appropriate information sheets and consent documents for the research process, designing manuals in terms of language clarity) [21, 29, 33, 34, 42].

The use of PPI in fundraising has also been reported [29, 33]. Six reviews analyzed PPI’s impact on research data [28, 29, 34, 37, 40, 42]. Five studies found an increased depth of data and understanding when using PPI [28, 29, 34, 40, 42], two studies noted PPI use led to more meaningful and valid results [40, 42], and five reviews showed that PPI determined studies’ research question(s) [25, 27, 29, 34, 37, 42].

#### Impact on academic researchers and academic institutions

Two reviews indicated that PPI use changed attitudes toward PPI and collaborating with researchers [27, 29]. Burton et al. found that researchers had built a PPI network that could be used in future studies [33]. Another review revealed that researchers perceived a validation of their research ideas [37]. Three reviews found that researchers can gain a broader understanding of people with lived experiences and broaden their perspectives [28, 37, 42]. Two reviews reported reciprocal learning among academic researchers and involved co-researchers [36, 42]. Sheikhan et al. noted a perceived positive change in organizational culture and a shift in power dynamics [42].

#### Impact on co-researchers

The positive aspects mentioned in seven reviews were: PPI provided a sense of purpose, pride, and gave people a voice [28, 29, 33, 40, 42]; social factors such as reduced isolation and perceived stigma [28, 42]; PPI was recognized as empowering, thus increasing autonomy [27, 29, 36, 38, 40, 42]; mutual learning and skill building [29, 42]; and co-researchers felt they were contributing to improving the care of their peer group [28, 29, 42].

#### Negative impact

Kowe et al. reported the negative impact of involving co-researchers [37], showing that PPI can lead to lower data quality, emotional burden for academic researchers, unsatisfactory reliability of the results, and changes in planned research [37]. Meanwhile, Carroll et al. reported the risk for including only co-researchers who are sympathetic toward the research goals of the academic researchers and who share their point of view [21].

### Discussion

This scoping review provides an overview of PPI in clinical and basic psychiatric research. Twenty reviews comprising 429 studies were included. The 20 reviews analyzed were spread over 15 years, with most being published in 2023 (five reviews) and 2017 (three reviews). The results of the scoping review and the increasing number of publications concerning PPI suggest an increasing recognition of PPI’s importance in this area. The results also suggested that PPI can improve the relevance and quality of research by including the perspectives of patients and the public.

However, the effects reported in the included studies were mostly anecdotal reports on PPI’s influence. Although these findings highlight the practical benefits and enriching perspectives that PPI can bring to scientific research, there remains a lack of rigor and robustness in systematically developing, evaluating, and implementing PPI in this field. Thus, future research should integrate these anecdotal insights with further empirical data to derive clear, robust implications for practice.

The results showed that PPI is also associated with many challenges and obstacles. These include insufficient resources and researchers trained on PPI use. PPI approaches vary considerably, leading to inconsistencies in the quality and depth of involvement. These findings highlight the need for clear guidelines and standards.

### Preconditions and aims

This review highlights that PPI is used in psychiatric research to pursue various aims. The individual requirements and objectives identified are generally intended to improve research and healthcare but remain vague. However, regarding other PPI guidelines, a standardized framework that coherently integrates PPI’s various objectives and methods, specifically for basic and clinical psychiatric research, is lacking. This scoping review’s results largely cover the aspects mentioned in other guidelines, albeit at a different, more basic level, and only fragmentarily.

When comparing the present scoping review’s results with the National Institute for Health and Care Research policy documents related to PPI in public health research, a similar diversity of purposes and aims for PPI becomes apparent [9]. Russel et al. emphasized that involving patients and the public in the entire research process leads to more relevant, reliable, and practically applicable research findings [9]. These documents highlight instrumental benefits, such as improving the quality and methods of research, and democratic goals, such as promoting participation and transparency.

Simultaneously, democratic objectives were addressed, albeit to a lesser extent. For example, Reyes et al. and Stacciarini et al. emphasized PPI’s role in understanding the (minority) communities’ needs, which promotes a more inclusive and representative approach to research [26, 41]. Jilani et al., whose handout referred to the participation of older people in research, emphasized the importance of clear aims for PPI, as these are prerequisites for evaluating PPI [47]. Clear aims and framework conditions can also prevent conflict among participants [47]. This is not directly addressed in the papers included in this scoping review, but is important, especially in research on people with cognitive impairments.

### Methods

The most commonly used PPI formats include focus groups, interviews, workshops, and advisory boards. However, not every method is suitable for actual involvement. Participants were more objects of study than partners in the research processes of four studies [25, 26, 29, 35]; this lack of real participation is not only due to participants’ underlying conditions. Only two studies involved individuals with dementia or intellectual disabilities [29, 35]. Particularly, when dementia or intellectual disability is progressive, the possibility of researchers and co-researchers working on an equal footing is limited [28–30, 33, 47, 48]. Others reviews reported the involvement of people with dementia, using methods such as focus groups, advisory boards, interviews, or workshops [31, 33, 35, 37]. Similarly, these methods do not guarantee genuine PPI on an equal footing, but they offer participants many more opportunities to participate and express their contributions. Therefore, we concluded that participants’ underlying conditions did not comprise a limiting factor for PPI. When methods are adapted, trained researchers and the target group’s specific needs are addressed in the research process (e.g., extra time and sensible moderation), which can also contribute to research. Another important point in our review is the adaptation of working materials and methods to the needs of the involved group. In our review, we compiled several adaptions, such as visual adaptions (large font, black print on colored paper, and visual prompts) and linguistic adaptions (avoiding jargon and reducing abstract language). These recommendations were not found in reviews or manuals on PPI used in health-service research. Thus, this point is either unique to PPI in psychiatric research or is not mentioned as a special point in other reviews.

Only one of the reviewed articles reported financial compensation [37]. Four reviews reported this point [27, 29, 34, 37, 42]. Fox et al. also reported funding for patient engagement in very few studies: only two reported financial compensation for the patients involved [49]. In a review by Souleymanov et al. about involving people who use drugs [27], fair compensation was the most discussed ethical issue [27]. Financial aspects were addressed as a burden when using PPI in research, such as for additional training [9, 11, 24, 47, 48, 50, 51]. Financial or other forms of compensation for participants is recommended in PPI manuals [52, 53]. Financial compensation should be considered from the beginning and included in research planning [29].

However, the various stages of involvement have not been consistently reported. Different systems for classifying PPI were used: Arnstein’s classification [37, 43] Roger Hart’s ladder of participation [36], Wright’s stage model of participation [22], and Sellars et al.’s levels of involvement [22, 44]. The 8-stage ladder of participation by Roger Hart and the 5-stage level of involvement adapted from Sellars are models for the participation of young people. Arnstein’s classification (8 levels) and Wright’s levels of participation (9 levels) are models for citizen participation.

However, particularly for appropriate PPI implementation and evaluation, information about the stages of involvement can be an important factor. The use of a participatory stage model can also help participants decide whether they want to engage in PPI and to what extent. From the researchers’ perspective, clarity regarding the level of participation can avoid misunderstandings and frustrations.

### Evaluation

In our review, there was limited information regarding evaluation methods, but there were almost exclusively positive effects on the research itself, academic researchers, and co-researchers. Only three reviews provided detailed information regarding the evaluation methods used [32, 34, 38]. The other articles included in our review discussed limited formal evaluations [21, 34]. Thus, PPI’s reported positive impact on research, academic researchers, and co-researchers should be considered with caution.

Thus, little is known about PPI’s impact on power imbalances between academic researchers and co-researchers [54]. If co-researchers are under pressure to make a measurably successful contribution, this could have a negative impact on the important factor of trust. The lack of standardized methods for measuring the impact as well as generally accepted relevant endpoints pose a problem when comparing and comparing the impact. On the other hand, the measurement of impact must also be based on the aims for which PPI was used [54]. Russel et al. criticized the predominant focus on PPI use to obtain measurable outcomes and argued that PPI is an end in itself and part of a research process [54].

Furthermore, there is a need for research that acknowledges and reports on PPI’s potential negative impacts, that is only reported in one review [37, 54]. They posited lower data quality, emotional burden on academic researchers, lack of reliability of the results, and changes in planned research as negative effects [37].

Blackburn et al. investigated PPI’s evaluation and its impact on (non-psychiatric) research under different health conditions in a mixed-methods study [51]. They used Boote et al.’s quality indicators, including “Roles of public involvement members are documented,” “Researchers training needs for public involvement are met,” and “Contribution of PPI included in research reports and papers” [55]. Blackburn et al. noted PPI’s positive impact on commitment to study design and writing patient information but a weaker impact on carrying out the research itself [51]. By contrast, Brett et al. reported PPI’s positive impact on involvement at all stages of the health and social research process [10]. Brett et al. reported the positive impacts of developing research topics, identifying patient-relevant outcomes, and improving patient information and questionnaires [10]. Some of the involved patients were engaged as interviewers, which had a positive impact on the dissemination of the findings [10]. Blackburn et al., Boada et al., Fox et al. and Brett et al. mentioned PPI’s positive effect on the recruitment of participants or research partners [10, 28, 33, 34, 42, 47–49, 51].

Recruitment and participation rates are objectively measurable; therefore, we can assume that they have a relatively stable and positive impact on PPI in different research areas. Brett et al. reported PPI’s “challenging impact” [10]. Particularly, in the early stages of a research project, barriers such as tensions between the academic criteria of good-quality research and co-researchers’ perspective on the appropriateness of the methods or PPI’s tokenistic use in research can arise [10]. The risk of tokenistic PPI use was also mentioned by Hawke et al. and Carroll et al. [21, 39].

Our review identified trust-building and a strong relationship between academic researchers and co-researchers. This point was emphasized in 10 of the 20 included reviews. The importance of this aspect is supported by various reviews of PPI in various research areas [10, 47–49, 51, 56]. Therefore, we conclude that trust- and relationship-building may be the main factors for successful PPI in every research area. This finding is particularly relevant in the context of psychiatric patients and their loved ones, given the perceived stigma associated with psychiatric conditions [4, 12]. This stigma has also been addressed in reviews by Jakobsson et al., Totzeck et al., Di Lorito et al. and Sheikhan et al. [22, 28, 40, 42]. It is important to highlight Jakobsson et al.’s findings regarding the need to carefully explain data storage when working with individuals who have experienced psychotic episodes [40].

The importance of defining co-researchers’ roles in the research process has been emphasized by Brett et al., Russel et al., Berns et al., Fox et al., and McCarty et al. [9, 10, 24, 49, 54, 57]. Thus, this appears to be a central point that can be transferred across all research areas. Blackburn et al. suggested that these roles should be documented [51]. The recommendation for PPI roles to remain flexible, made by Di Lorito et al. and Totzeck et al., is supported by Skovlund et al. and Brett et al. [10, 22, 28, 29, 56]. In these studies, the importance of adapting involvement and engagement in the research process is highlighted [10, 56]. Defining roles and maintaining flexibility are not exclusive. At the beginning of a research project, it is possible to explore participants’ and the public’s willingness and ability to be involved in the research and evaluate this continuously to adapt these roles. Di Lorito et al. emphasized that in the course of the research process, co-researchers may become more confident and willing to participate [29]. This issue may be related to a power imbalance. The power imbalances addressed in the five reviews appear to be a recurrent theme in studies involving psychiatric patients [28, 30, 38, 40, 42]. In other reviews on PPI in health and preclinical research, these imbalances are not represented. Only Russel et al. mentioned that power imbalances are a challenging issue in PPI use [54]. It is possible that addressing power imbalances is important for cooperation among psychiatric patients. This may be due to the perceived stigma associated with psychiatric diseases [4, 58, 59].

In our view, in preclinical research, language should be adapted to effectively communicate with the people involved. Given that trust- and relationship-building are key factors in successful PPI, effective communication is essential. Productive trust-building communication can only occur if both sides understand each other, which implies that academic researchers must communicate in a way that is accessible to the participants. Maccarthy et al. identified communication difficulties, including academic researchers’ concerns regarding communicating appropriately with those involved, as key factors in academic researchers’ implementation of PPI [24]. Russel et al. pointed out that academic researchers are reluctant to adapt to working practices [54]. This reluctance was also identified in three other papers [21, 38, 42].

The key barriers to PPI implementation in research include budgeting and planning. Compensation for the co-researchers’ time and travel should be budgeted at the beginning of the research project. Additional costs may arise from training sessions or team-building events. Furthermore, extra time may be needed for the research project due to additional meetings or adapted methods to meet participants’ needs, which can incur further costs. These barriers have also been noted for PPI implementation in other research areas [24, 49, 56, 60]. Blackburn et al. identified time as the “main cost” for researchers [51]. Additional time and financial burdens should be considered in project and financial planning.

Appropriate PPI planning should encompass the use of guidelines and the proper recruitment of co-researchers. Moreover, factors such as cultural characteristics, age, gender, and underlying conditions should be considered. This is supported by Skovlund et al. and Fox et al. [49, 56]. Brett et al. pointed out the challenges of recruiting hard-to-reach groups such as older people or people with disabilities [10]. Burton et al. mentioned the difficulties in managing large PPI groups [33]. Skovlund et al. recommend engaging a balanced number of patients, the public, and other researchers [56].

The use of PPI guidelines can support PPI implementation in research processes, as supported by a survey by Maccarthy et al. [24]. From our perspective, the use of structured guidelines can help overcome barriers such as power imbalances or rigid roles and raise awareness of PPI’s pitfalls.

### Training

The need to train academic researchers, involved patients, and the public is crucial in successful PPI [22, 27, 29, 33, 37, 39, 42]. This training should entail technical, research [22, 27, 29, 37, 39], and communication [29] skills. Such training’s importance was supported by Blackburn et al., Fox et al., and Brett et al. [10, 49, 51]. As noted by Hawke et al., Brett et al. reported that training can help avoid tokenism and maximize authentic PPI [10, 39]. Similar to our results, the costs of training and a longer research duration were discussed by Brett et al. [10]. Meanwhile, Skovlund et al. and Maccarthy et al. highlighted the need for training in achieving successful PPI [24, 56].

### PPI in basic psychiatric research

While PPI has become established in many areas of clinical research, it is still comparatively rare in the field of basic research. Since preclinical research, such as laboratory-based research, takes place in rather inaccessible environments, PPI is a particular challenge here and should therefore be discussed separately. PPI in preclinical research generally aligns with the findings of reviews from health service research. Involvement can extend to co-authorship and the dissemination of findings [21]. Nunn et al. reported involvement in genomic research at different stages of the research process: implementation, dissemination, evaluation, design, data analysis, identification of topics, and different common methods of involving people, such as format focus groups, interviews, workshops, and feedback groups [60]. Of the 32 studies summarized in this review, four reported PPI at every stage of the research process [60]. Fox et al. reported the predominant use of PPI in laboratory research, in training and priority settings [49]. However, in laboratory research, people’s involvement in the study design and the dissemination of results is possible [49]. Sole involvement in data collection or analysis was not observed [49]. Fox et al. argued that this is because laboratory research is not patient-oriented and access for patients to animal laboratory facilities is limited [49]. As with the other reviews included in our study, the most common formats of involvement in preclinical psychiatric research were focus groups, interviews, workshops, and feedback groups [21]. Nunn et al. and Fox et al. also identified workshops, focus groups and interviews as the common methods involving people in genomic and laboratory research [49, 60]. In genomic research, formal groups such as steering committees or public events like debates and workshops were more frequently used [60]. Additionally, surveys, online tools (e.g., social media), newsletters, consultations, and citizen science were used [60].

As in other research areas, trust and strong relationships are the key facilitators of successful PPI in preclinical research [21]. This is supported by a scoping review by Fox et al. and Maccarthy et al. [24, 49]. Nunn et al. emphasized the need for establishing trust among stakeholders in the process of managing conflicting interests [60].

Facilitators not mentioned by Carroll et al. were found in Fox et al.’s review of PPI in laboratory research; these included financial compensation for those involved, proper planning, and a structure to support patients’ commitment [49]. However, Carroll et al. identified barriers less frequently mentioned: skepticism from academic researchers and concerns related to the required long-term commitment [21]. Maccarthy et al. reported academic researchers’ reluctance to adopt PPI [24]. Researchers have expressed concerns about communicating appropriately and managing the underlying conditions of the patients involved [24]. Research areas in this survey extended from rheumatic diseases to diabetes and different cancers [24].

Carroll et al. highlighted recruitment as a practical barrier [21]. Insufficient diversity in recruiting can lead to a lack of representativeness by including only co-researchers who are sympathetic to and share the views of the academic researchers [21]. Fox et al. also mentioned the inadvertent exclusion of target group members [49].

The need for PPI training for study participants, the public, and academic researchers was a focus in the research of Carroll et al., Fox et al., and Nunn et al. [21, 49, 60]. Maccarthy et al. indicated that this training could cover communication skills, presentation skills, and media training [24]. Nunn et al. did not specify the content of training [60]. Fox et al. only reported the need for training researchers, but not the co-researchers [49].

As our results show, PPI is applicable at different stages of preclinical research using different methods [21, 49, 60]. At certain stages of specific research design, such as data collection in laboratory research, there is insufficient PPI [49]. This could be due to skepticism on the part of the researchers, but also to certain legal conditions to which laboratory research is subject (e.g. Animal Welfare Experimental Animal Ordinance TierSchVersV).

Overall, the present scoping review’s results regarding PPI implementation in preclinical research did not differ strongly from those for PPI implementation in health service research. The results from Carroll et al. for the included psychiatric studies did not differ from the key points for implementing PPI in other than psychiatric research. It can be concluded that PPI Implementation is possible in preclinical psychiatric settings and associated with a positive impact for research.

### Strengths and limitations

To our knowledge, this scoping review is the first to systematically compile, analyze, and process evidence on PPI implementation in basic and clinical psychiatric research settings. Through this systematic compilation and embedding in an overarching project, namely, the DZPG, the results can be effectively implemented in practice. This scoping review was created with the help of PPI and was accompanied by a panel of experts and patient representatives with the support of the DZPG.

One of the primary limitations of our review was the potential for selection bias. Relevant reviews that were not included in the databases we searched may have been overlooked. Moreover, we excluded individual studies that were not included in the reviews. This is also reflected in the limitations of the included reviews: The search strategies employed by the included reviews could have led to selection bias [21, 27, 30, 34–37, 39–42]. Another issue highlighted by several authors is the inconsistent terminology used for PPI [21, 30, 35, 37, 41, 42], which may also contribute to selection bias.

Additionally, the broad range of reviews introduces a high degree of methodological and population-related heterogeneity. These variations make it difficult to compare, synthesize, and generalize the results. This issue was directly addressed by Cowdell et al. and Di Lorito et al. [28, 35] and is indirectly relevant to all included reviews, as they focused on specific populations, disease conditions, or cultural contexts, thus limiting the breadth of applicability. However, the value of qualitative research and other forms of reporting in this context should be recognized and valued, despite their limited generalizability, as they provide in-depth insights into complex processes (e.g., PPI) that quantitative methods might not capture. This was emphasized by Carroll et al., who described reports on preclinical research more as reflections of the process than as classical trial reports.

Because this was a scoping (rather than systematic or realist) review, we did not conduct a formal quality assessment of the included studies nor have we gone deeper into the analysis to identify causes of barriers and implications. This is an inherent limitation of scoping reviews, which typically do not involve critical appraisals. However, our results should be interpreted with caution, as the methodological quality of the underlying studies may vary significantly. Notably, some included reviews did not assess the quality of the included studies, further compounding this issue. Some of the reviews mentioned methodological weaknesses, methodological diversity, and the challenge of separately measuring PPI’s separately within a process [21, 22, 28, 29, 31, 40]. These issues complicate the comparison and synthesis of findings, as emphasized by Jakobsson et al. [40].

Several reviews cited the general lack of publications as a limitation [21, 25, 28, 35]. This shortage reflects an underreporting problem and disparities in the reporting of PPI-related research, as noted by Valdes et al., Ragavan et al., Miah et al., Hawke et al., and Totzeck et al. [22, 32, 34, 36, 39]. Some reviews have suggested that publication bias may arise because researchers are more likely to report on studies in which PPI interventions were successful [35, 39]. This underscores the need for standardized reporting practices and raising awareness that unsuccessful or negative results must also be published, in line with good scientific practice. Failure to report these outcomes hinders one’s ability to learn from failures and identify potential pitfalls. Furthermore, it should be emphasized that the lack of publications culminates when looking specifically at preclinical research, as only Carroll et al. were able to identify the literature on PPI in this area. Presumably, there may be a lack of awareness among researchers in this field regarding PPI being feasible and necessary, since this type of research lays the foundation for all subsequent stages of research and should therefore be relevant and comprehensive from the start.

From the perspective of the included reviews, recurrent publication and selection biases, along with other methodological weaknesses in the reviewed literature, suggest that the current presentation of the evidence base may be distorted by these biases, ultimately affecting the validity and reliability of the research outcomes.

## Conclusion and implications

The results of this scoping review show PPI’s increasing importance in psychiatric research. However, they also show that research and practice on PPI in this area are still in the early stages. Further investigations into PPI are needed, especially regarding basic research and PPI’s implementation and impact. Initial recommendations for practice and future research can be derived from the results and serve as a framework for the DZPG to realize the goal of integrating PPI in psychiatric research.

### General conclusions for practice

General recommendations for practice can be derived from this review’s results, which can lead to the establishment of PPI structures for psychiatric clinical and basic research. Our review aims to strengthen the practice of PPI and ensure that the perspectives of patients and the public can be integrated to ultimately achieve the goal of patient orientation and relevance in evidence-based medicine. Moreover, the results will be validated with experts and developed further over the course of our future research. The golden rules for practice (clinical and basic research) derived from the results are as follows:

1. Foster a culture of collaboration and mutual respect between researchers and PPI participants.
2. Provide adequate resources and support for PPI activities, including funding and training programs.
3. Develop clear guidelines and standards for the management of PPI to ensure consistency and quality.
4. Develop a willingness to integrate PPI into all phases of research, from planning to the dissemination of results.

### Recommendations for future research

Based on the identified knowledge gaps and challenges, future research should consider the following aspects.

1. Developing and implementing standardized methods and clear objectives for assessing PPI’s quality in research.
2. Investigate PPI’s long-term impact on research and health outcomes, not with the aim of proving its efficacy but aiming to make PPI as effective as possible for patients’ benefit.
3. Develop, test, and evaluate effective training programs for researchers and PPI participants to improve the quality of participation.
4. Analyze the barriers and facilitators to effective PPI implementation in different contexts.

## Electronic supplementary material

Below is the link to the electronic supplementary material.

Supplementary Material 1

Supplementary Material 2

Supplementary Material 3

Supplementary Material 4

Supplementary Material 5

## Data Availability

No datasets were generated or analysed during the current study.
