# Supplementary material for: Patient and public involvement in basic and clinical psychiatric research: a scoping review of reviews
Source: BMC Psychiatry. 2025 Mar 25;25:283. doi: 10.1186/s12888-025-06608-7 (PMC11938574; doi:10.1186/s12888-025-06608-7)
Supplement: Supplementary file 1 — Supplementary Material 1 [file 12888_2025_6608_MOESM1_ESM.docx]

**Supplementary file 1: Search strategies**

**(A OR A2) AND (((B OR D1) AND C2)OR B2 OR C1) 2008 - 2023**

**Cochrane Library**

| A | ("patient involvement") OR ("Patient & Public Involvement") OR ("Patient and Public Involvement") OR ("Public and patient partnership") OR ("Public and patient involvement") OR ("peer research") |
| --- | --- |
| A2 | (“community-based research”) OR (“participatory research”) OR empowerment OR (“capacity-building”) OR (“collaborative research”) |
| B | psychiatr* |
| B2 | (anxiety) OR (phobi*) OR ("post-traumatic stress disorder") OR (PTSD) OR ("eating disorder") OR (anorexia) OR (bulimia) OR (depression) OR (depress*) OR ("obsessive compulsive disorder") OR (OCD) OR (psychosis) OR (bipolar) OR (mania) OR (schizophrenia) OR (adhd) OR ("attention deficit hyperactivity disorder") OR ("substance abuse") OR (addict*) OR (dementia) OR ("Alzheimer disease") |
| C1 | (drug NEXT/1 development) OR (chemical NEXT/1 analysis) OR (chemistry NEXT/1 techniques) OR (genetic NEXT/1 research) (genom NEXT/1 research) OR (neuroanatom*) OR (behavioral pharmacolog*) OR (chemistr* AND (neuro* OR biol*)) OR (metaboli* NEXT/1 patholog*) OR (endocrinolog*) OR (MRI OR ("magnetic resonance imaging")) OR (MRS OR (magnetic NEXT resonance NEXT spectroscop*)) OR (biomedicine) OR (biomedical NEXT/1 science) OR (simulation AND (acustic OR visual)) |
| C2 | ("clinical research") OR ("preclinical research") OR ("basic research") OR ("basic scientific research") OR ("laboratory research") |
| D1 | ("mental health") |

**Embase & PsycInfo**

| A | ("patient involvement") OR ("Patient & Public Involvement") OR ("Patient and Public Involvement") OR ("Public and patient partnership") OR ("Public and patient involvement") OR ("peer research") |
| --- | --- |
| A2 | ("community-based research") OR ("participatory research") OR empowerment OR ("capacity-building") OR ("collaborative research") |
| B | psychiatr* |
| B2 | (anxiety) OR (phobi*) OR ("post-traumatic stress disorder") OR (PTSD) OR ("eating disorder") OR (anorexia) OR (bulimia) OR (depression) OR (depress*) OR ("obsessive compulsive disorder") OR (OCD) OR (psychosis) OR (bipolar) OR (mania) OR (schizophrenia) OR (adhd) OR ("attention deficit hyperactivity disorder") OR ("substance abuse") OR (addict*) OR (dementia) OR ("Alzheimer disease") |
| C1 | ("drug development” OR (drug ADJ1 development*)) OR ("chemical analysis" OR (chemistry ADJ1 techni*)) OR (gene* ADJ1 research) OR (neuroanatom*) OR ("behavioral pharmacolog*") OR (chemistr* ADJ1 (neuro* OR biol*)) OR ("metaboli* patholog*") OR (endocrinolog*) OR (MRI OR ("magnetic resonance imaging")) OR (MRS OR ("magnetic resonance spectroscop*")) OR (biomedicine OR (biomedical ADJ1 science*)) OR (simulation ADJ1 (acustic OR visual)) |
| C2 | ("clinical research") OR ("preclinical research") OR ("basic research") OR ("basic scientific research") OR ("laboratory research") |
| D1 | ("mental health") |

**Psyndex**

| A | ("patient involvement") OR ("Patient & Public Involvement") OR ("Patient and Public Involvement") OR ("Public and patient partnership") OR ("Public and patient involvement") OR ("peer research") |
| --- | --- |
| A2 | ("community-based research") OR ("participatory research") OR empowerment OR ("capacity-building") OR ("collaborative research") |
| B | psychiatr* |
| B2 | (anxiety) OR (phobi*) OR ("post-traumatic stress disorder") OR (PTSD) OR ("eating disorder") OR (anorexia) OR (bulimia) OR (depression) OR (depress*) OR ("obsessive compulsive disorder") OR (OCD) OR (psychosis) OR (bipolar) OR (mania) OR (schizophrenia) OR (adhd) OR ("attention deficit hyperactivity disorder") OR ("substance abuse") OR (addict*) OR (dementia) OR ("Alzheimer disease") |
| C1 | (drug development/exp OR (drug W/1 development*)) OR (chemical analysis/exp OR (chemistry W/1 techni*)) OR (gene* NEXT/1 research) OR (neuroanatom*) OR (behavioral pharmacolog*) OR (chemistr* W/1 (neuro* OR biol*)) OR (metaboli* patholog*) OR (endocrinolog*) OR (MRI OR (“magnetic resonance imaging”)) OR (MRS OR (“magnetic resonance spectroscop*”)) OR (biomedicine/exp OR (biomedical W/1 science*)) OR (simulation W/1 (acustic OR visual)) |
| C2 | ("clinical research") OR ("preclinical research") OR ("basic research") OR ("basic scientific research") OR ("laboratory research") |
| D1 | ("mental health") |

**Web of Science**

| A | ("patient involvement") OR ("Patient & Public Involvement") OR ("Patient and Public Involvement") OR ("Public and patient partnership") OR ("Public and patient involvement") OR ("peer research") |
| --- | --- |
| A2 | ("community-based research") OR ("participatory research") OR empowerment OR ("capacity-building") OR ("collaborative research") |
| B | psychiatr* |
| B2 | (anxiety) OR (phobi*) OR ("post-traumatic stress disorder") OR (PTSD) OR ("eating disorder") OR (anorexia) OR (bulimia) OR (depression) OR (depress*) OR ("obsessive compulsive disorder") OR (OCD) OR (psychosis) OR (bipolar) OR (mania) OR (schizophrenia) OR (adhd) OR ("attention deficit hyperactivity disorder") OR ("substance abuse") OR (addict*) OR (dementia) OR ("Alzheimer disease") |
| C1 | (drug NEAR/1 development) OR (chemical NEAR/1 analysis) OR (chemistry NEAR/1 techniques) OR (genetic NEAR/1 research) (genom NEAR/1 research) OR (neuroanatom*) OR (behavioral pharmacolog*) OR (chemistr* AND (neuro* OR biol*)) OR (metaboli* patholog*) OR (endocrinolog*) OR (MRI OR ("magnetic resonance imaging")) OR (MRS OR ("magnetic resonance spectroscop*")) OR (biomedicine) OR (biomedical NEAR/1 science) OR (simulation AND (acustic OR visual)) |
| C2 | ("clinical research") OR ("preclinical research") OR ("basic research") OR ("basic scientific research") OR ("laboratory research") |
| D1 | ("mental health") |
