# Supplementary material for: Patient and public involvement in basic and clinical psychiatric research: a scoping review of reviews
Source: BMC Psychiatry. 2025 Mar 25;25:283. doi: 10.1186/s12888-025-06608-7 (PMC11938574; doi:10.1186/s12888-025-06608-7)
Supplement: Supplementary file 2 — Supplementary Material 2 [file 12888_2025_6608_MOESM2_ESM.docx]

**Supplementary file 2: Characteristics of reviews**

| Study ID | Number included studies | Study design paper | Methodology included studies | Period | Countries included papers | Who was involved? |
| --- | --- | --- | --- | --- | --- | --- |
| Burton_2019 | 16 | Scoping review | Qual & quant | 2018 - 2019 |  | PLWD and caregiver, public |
| Carroll_2022 | 2 from 9 | Scoping review | Quant | 2011, 2018 | Netherlands, UK | People with autism, family/people who worked with children with autism; patients with schizophrenia |
| Cowdell_2020 | 9 from 48 | Scoping review | Qual, process evaluation, mixed methods | 2012 - 2018 | Australia, UK, Canada, USA, Netherlands | PLWD, residents, carers of PWLD, |
| Crocker_2018 | 3 oder 4 von 26 | Systematic review & meta analysis |  | 2003 - 2015 | USA, UK | People aged ≥65 with symptoms of depression, anxiety, at-risk drinking, Gulf War veterans with fatigue, musculoskeletal pain, cognitive complaints, Patients with moderate/severe Alzheimer’s disease (treated with donepezil for ≥3 months) |
| Di Lorito_2017(1) | 7 | Synthesis of the evidence | Qual & quant | 2003 - 2015,  no restrictions on publication date |  | PLWD, older people, mental health service users, people with learning difficulties |
| Di Lorito_2017(2) | 13 | Systematic review | Case reports & fesability studies | 1996 - 2016 | United Kingdom, Australia, New Zealand, USA | Adults with intellectual disability |
| Florence_2023 | 20 | Scoping review | Qual & mixed | 2009 - 2021 | Brazil (nearly half of the studies conducted in partnership with a Global North country) | Mental health service users; family members; providers |
| Hawke_2023 | 49 | Scoping review |  | 2017 - 2022 | UK, Canada, Australia, New Zealand, USA, Norway, Ireland, Germany, Sweden | PWLE of mental health or substance use |
| Jakobsson_2023 | 15 | Scoping review | Qual, quant descriptive studies | 2004 - 2020 | UK, Ireland, Denmark, Canada, Indonesia, 15 Countries | PWLE of psychosis |
| Kowe_2022 | 9 | Systematic review | Qual, mixed methods, descriptive | 2000 - 2020 | UK, USA, Belgium | PLWD or mild cognitive impairment |
| Miah_2019 | 20 | Scoping review |  | 2000 - 2018 | UK, Netherland | PLWD and/or care partners |
| Ragavan_2018 | 20 | Systematic review | Community-based research interventions | 1996 - 2016 | USA | Domestic Violence Survivors, Other community partners |
| Reyes_2023 | 163 | Scoping review | Qual, quant, mixed methods, process articles | 1995 - 2023 | UK, North America, Oceania, Europe, Asia, Other (including multiregion) | PLWD, people from oppressed ethno-racial groups, who are primarily affected by ADRD, practitioners, caregivers |
| Schilling_2017 | 9 | Literature review | quant | 2007 - 2017 | UK | PWLE of old-age-related condition, carers |
| Sheikhan_2023 | 61 | Scoping review | Process articles, qual, mixed, quant, commentary, Case study | 2012 - 2022 | Canada, USA, UK, Australia, New Zealand, Ireland, Germany, Norway, Sweden | PWLE of substance use and mental health conditions |
| Souleymanov_2016 | 25 | Scoping review |  | 1985 - 2013 | Canada, USA., UK, Australia | people who use drugs |
| Stacciarini_2009 | 5 | Literature review | Quant, qual | 1990 - 2008 | USA |  |
| Stacciarini_2010 | 20 | Literature review | Quant &qual | 2003 - 2010 |  |  |
| Totzeck_2024 | 19 | Systematic review | Mixed, qual | 2000 - 2023 | UK, Irland, Canada, USA, Australien, Schweiz, Chile, Schweden | CYP (age range: 10–26 years) |
| Valdez_2020 | 15 | Systematic review | Quant, qual, mixed, photovoice | 1998 - 2018 | USA, Canada, Bosnia Herzegovina | Youth of vulnerable backgrounds (i.e., rural, indigenous, street involved, refugee, conflict survivors) |

USA=United States of Amerika, UK=United Kingdom, PLWD= people living with dementia, PWLE=people with living experience, ADRD=Alzheimer's Disease and Alzheimer's Disease Related Dementias, CYP=children and young people
