# Supplementary material for: Patient and public involvement in basic and clinical psychiatric research: a scoping review of reviews
Source: BMC Psychiatry. 2025 Mar 25;25:283. doi: 10.1186/s12888-025-06608-7 (PMC11938574; doi:10.1186/s12888-025-06608-7)
Supplement: Supplementary file 3 — Supplementary Material 3 [file 12888_2025_6608_MOESM3_ESM.docx]

**Supplementary file 3: Outcomes „preconditions & aims” and “methods”**

| **Study ID** | **Preconditions & aims** | **methods** | | | |
| --- | --- | --- | --- | --- | --- |
|  |  | PPI Format | Financial compensation | PPI Implementation | Max. stage of involvement |
| Burton_2019 |  | Advisory committees |  |  | Half of the studies co-authored by PPI representatives |
| Carroll_2022 | **Objectives related to the research process** 1 Study: funders mandate, integration of various knowledge  **Objectives related to the results of the research** 1 Study: identify what is acceptable and unacceptable, assess the diversity of the community's views | Feedback on material (directly or via email), interviews, focus groups, jointly delivered conference workshop |  | Using framework for PPI | Study 1: designing and managing  Study 2: identifying and prioritizing, disseminating |
| Cowdell_2020 | **Objectives related to the research process**: select content and language, refine content and evaluate the acceptability and utility, address limitations of previous online interventions | Focus group/talking circle, Interviews, workshops, participant observation, prototypes tested, consultations, interactive web tool |  |  | Rarely engaged in planning/dissemination, predominantly only provision of data, no real participation |
| Crocker_2018 | **Objectives related to the research process**: enhance recruitment and recruitment rates, retention of minoritys, improve informed consent | Advisory board meetings, focus groups |  | Discussed at bimonthly consumer advisory board meetings. |  |
| Di Lorito_2017(1) |  |  |  |  |  |
| Di Lorito_2017(2) | **Objectives related to the research process**: ensure the project is appropriateness, accessibility and sensitivity on research material e.g. study protocol, information sheets, consent forms, questionnaires.  Advise on relevant study outcomes. | Interviews, focus group, observations, video-recordings, personal diaries, field notes | Discussed in 2 papers | PAR, Throughout the study, consulted every 3 months Type of PPI:  Consultative (5)  Advisory (2)  Collaborative (2)  Co-research (3) | 5 Studies: consultation/reference groups  2 Studies: advisory groups  3 Studies: PPI members acted as equal partners in collaboration and production of research  2 Studies: PPI members acted as co-researchers conducting interviews alongside academics. |
| Florence_2023 |  | Focus groups, workshops, research meetings, co-leading intervention groups, participants were lecturers at university, users wrote about research experience. |  |  | 2 Studies: participants involved in all stages of research.  No co-authorship in any study. |
| Hawke_2023 |  |  |  |  |  |
| Jakobsson_2023 |  | Collaborative workshops, reference/steering groups, consensus seeking with service users, co-research |  | Most of the studies used continuous reflections and ongoing dialogue to support an iterative process of collaboration. | Application of INVOLVE features on included studies: except 1 study all failed to report ground rules at the onset of the research project, |
| Kowe_2022 |  |  | 3 Papers: financial compensation for co-researchers | 2 Publications: training for co-researchers; 1 Paper: training for lead researcher, 1 Paper: training for all | Degree of participation according to Arnstein (1969) Lowest: tokenism (2) Highest: delegated power (2) |
| Miah_2019 | **Objectives related to the research process**: identifying & prioritizing research questions (n = 4), research design (n = 5), conducting research (n = 8), data analysis & interpretation (n = 3) | Workshops, drop-in sessions, (individual) meetings, modified Delphi process combined with a consensus conference, anonymous reader consultation, participatory approach, interviews, focus groups, questionnaire, voiceover group, PPI-event. | Discussed in one paper, not further elaborated. | PPI | Participants involvement in all stages of research process |
| Ragavan_2018 |  |  |  | CBPR | Guided project design, reviewers of project materials, 10 articles contained authors with nonacademic affiliation |
| Reyes_2023 | **Objectives related to the results of the research:**   - Enhancing quality of dementia care (majority), - Increase awareness of dementia + dementia service - Understand environmental and place-based contexts - Understanding lived experience   **Objectives related to research in general:**   - Demonstrate the feasibility of partnering - Improve outreach to, and research participation | Varied widely: participation ranged from traditional participant subject to being part of a steering committee or feedback group. |  | - Consensus model: every participant has an equal vote. - Decentralized model: subcommittees with representatives responsible for specific aspects. - Many studies use "participatory" without definition/description.   Participatory research methods:  action research n=54, PAR n=47, CBPR n=33, Participatory research n=15, Co-research n=8, Other n=6 | Up to co-authoring. |
| Schilling_2017 | **Objectives related to the results of the research:** to enhance the connection to the target population,  **Objectives related to the research process:** documents & recruitment, the interview experiences and data, validity of results,  **Objectives related to research in general:** acceptability & feasibility of future research, improve the use of resources | Co-production in shared domicile, anonymous postal reader consultation, local reference group, workshop, core reference group, focus groups; Survey, co-research |  | PPI | Participants functioned as partners and shared control with researchers |
| Sheikhan_2023 |  |  | Discussed in 11 studies |  |  |
| Souleymanov_2016 |  | Training them as peer interviewers, training people as peer public health advocates (PHA) | 8/ 25 Studies explored ethics of compensation & incentive payments. 3 studies explored how compensating participants ( cash vs. other forms of compensation) improves quality of research & retention of people | CBPR | Active role in guiding, developing, and conducting the research |
| Stacciarini_2009 |  | Meetings, shared ownership of the data collected |  | CBPR | Shared ownership of the data collected, results of data analysis, interpretations & dissemination of findings. |
| Stacciarini_2010 | **Objectives related to the results of the research:** identifying communities’ needs  **Objectives related to research in general:** recognizing community members as vital collaborators in research efforts to address mental health issues | Large variety of methods for data collection were used (e.g., interviews, surveys, focus groups, activities involving the arts, stake holders’ discussions, and field notes). |  | CBPR |  |
| Totzeck_2024 |  | Advisory groups, focus groups, consultation groups or youth and expert references groups, forming small local groups instead of large online discussions |  | PPI approaches: YPAG n=5, PAR n=3, CBPR n=3 | Stage model of participation by Wright (2010)  n=3 studies stand out because they reached level 8, majority (n = 14) ranged between levels 5 (“Inclusion”) and 7 (“Partly authorized to make decisions |
| Valdez_2020 | **Objectives related to the research process:** aiming to conduct a community assessment of youth substance use, develop an intervention or program for substance use prevention  **Objectives related to the results of the research**: reduce and/or delay onset of substance use in youth  **Objectives related to research in general:** objectives related to the research process |  |  | YPAR (n=8) CBPR (n=4)  PYD (n=3) Hart’s Ladder of participation (n=1) (8 escalating degrees of participation), participatory intervention model (PIM) (n=1), having a formal/informal agreement regarding mutual decision-making about potential changes in research methods or focus | Nearly all studies: interpretation of research findings (n=13)   Formal or informal agreement regarding mutual decision-making about potential changes in research methods or focus (n=8)  Involving youth in the dissemination of research findings (n=14) |

Participatory action research (PAR), Community-based participatory research (CBPR), Patient and public involvement (PPI), PYD is an intentional approach that recognizes, utilizes, and enhances young people’s strengths. PYD is a framework that is compatible with PAR/CBPR, but PYD does not require a research component.
