# Supplementary material for: Patient and public involvement in basic and clinical psychiatric research: a scoping review of reviews
Source: BMC Psychiatry. 2025 Mar 25;25:283. doi: 10.1186/s12888-025-06608-7 (PMC11938574; doi:10.1186/s12888-025-06608-7)
Supplement: Supplementary file 4 — Supplementary Material 4 [file 12888_2025_6608_MOESM4_ESM.docx]

**Supplementary file 4: Outcome “evaluation”**

| **Study ID** | **Evaluation** | | | |
| --- | --- | --- | --- | --- |
|  | Method | Supportive factors | Barriers | Impact of PPI |
| Burton_2019 | 1 study: questionnaire | **Practical strategies:** adapting reading materials (e.g. large font, black writing on colored paper, pictures, word cards or story boards), use of role play, meeting in accessible & familiar environments, reimbursing for time and travel  **Strategies for effective meetings:** clear agendas & terms of reference, allowing extra time, sensitive facilitator to encourage comment, flexibility in the method of participation (e.g. face-to-face, telephone or e-mail), contact between meetings, skills of  co-researcher so they could choose specific tasks & support provided to members identified who wanted to participate in areas they lacked confidence, involving ‘community insiders’ to engage hard-to-reach groups, importance of good communication, building relationships & trust, creating an informal environment, providing feedback on how co-researcher influenced the research, train researcher to address the additional communication needs of PWLD | Challenges mostly from researcher; engaging PLWD and hard-to-reach groups was identified as a challenge; lack of engagement with people with severe dementia, difficulties recruiting any PLWD to an advisory committee; challenges in obtaining feedback from PLWD because of difficulties understanding & remembering survey questions; PPI representatives recruited from existing dementia research groups possibly not representative of the wider community; difficulties engaging health professionals & older adults without dementia who queried how the project was relevant to them; PLWD engaged in data analysis: not provided with training or, because of time, resources and perceived burden, not involved in selecting extracts for analysis; loss of connection to the study during ‘quiet times’ and frustratingly long waits for results; practical and financial arrangements were identified as the biggest challenge (untimely reimbursement, difficulties accessing training resources); large PPI group - difficulties managing expectations and relationships, and planning dates; cultural barriers (lack of funds for interpreters - meaning exclusion of non-English speakers) | **Impact on research:** increasing recruitment rates - especially hard-to-reach groups, lower attrition rates, fundraising for further research, improve research materials, interview questions & techniques, intervention manuals  **Impact on academic researcher & academic institutions:** researchers reported building up their PPI networks for future studies  **Impact on co-researcher & Community:** PPI provided sense of purpose, pride & giving people a voice, Co-researcher presented results on conferences, increased awareness of experiences of PLWD & dementia services, those who attended an event reported fewer negative views about dementia than those who did not |
| Carroll_2022 |  | Identify common ground between researchers and co-researcher, helped form a relationship between | Training on conducting PPI correctly is needed; PPI contributors' viewpoints may not be representative of an entire patient population; dialogue model for interaction between patients and researchers - but not necessarily suited for preclinical research because of long-term nature of the research; concerns around a lack of subjectivity by PPI contributors being incompatible with preclinical research; representativeness of viewpoints from PPI contributors of the general patient population; initial skepticism of preclinical researchers towards PPI, lack of training and awareness of PPI methodologies; suitability of PPI for preclinical research projects which progress slowly and require a long commitment from contributors; | **Impact on research:** jointly submitted action paper (recommendations for improving communication & interaction) |
| Cowdell_2020 |  |  |  |  |
| Crocker_2018 |  |  |  | **Impact on research**: changes in recruitment strategy, in 3 from 4 psychiatric related studies no significant higher enrolment rates for PPI interventions compared to non-PPI interventions |
| Di Lorito_2017(1) |  | **Training of peer researchers:** develop research skills & familiarize with project, develop confidence of doing research, become aware of own capacities & limits, training sessions should tailored to peer researcher's needs  **Defining involvement & roles**: open discussion about roles, discuss mutual responsibilities & expectations to develop a relationship of trust & mutual respect  **Strategies to address cognitive impairment**: using simple & jargon-free language, reducing abstract language or concepts, considering non-verbal language as a valid communication tool, avoiding making assumptions, resisting the temptation to finish peer researcher's sentences, maintaining a relaxed attitude  **Strategies to deal with memory difficulties**: using visual prompts (e.g. laminated cards), to aid peer researchers in interviews  **Location of involvement**: university environments may challenge individuals with cognitive impairments, who may find them more threatening than their homes or public spaces  **Emotional stress**: research team should pay attention to the risk of emotional overburden during involvement.  **Financially compensate peer researchers.** | **Selecting representative peer researchers:** may peer researchers does not mirror the diversity of the community of people with dementia  **Negotiating research power:** academics may reluctant to cede control to peer researchers and/or delegate more complex research tasks such as handling sensitive information or assessing capacity to consent  **Cognitive impairment and Memory difficulties:** should be addressed with adaptive materials and language adaptions  **Emotional stress:** emotional overburden that may affect the peer researchers' well-being during involvement.  **Resource implications:** increase research costs due to providing training, financially compensating peer researchers, paying support workers to accompany the peer researchers | **Impact on co-researcher & community:** social factors like reduced isolation & less perceived stigmatization, help to improving services for people with dementia, identity & improve life quality  **Impact on academic researcher & academic institutions:** wider understanding of people with lived experience  **Impact on research:** increasing recruitment rates, may increase the depth of data |
| Di Lorito_2017(2) |  | **Research training**: for co-researchers (reported in all the reviewed articles), aimed to develop technical skills (e.g. dealing with information sheets & consent forms, operating tape-recorders, taking notes, conducting interviews), developing relational skills, (e.g. learning how to be a good listener, relate to people with different background. Use user-friendly material.  **Team time together, even outside of research time**: e.g. informal chats, opportunity for co-researchers to share feelings around involvement & for academic researchers to develop a deeper understanding of the experience of living with intellectual disability  **Research roles:** defining the role of researchers & co-researcher, research roles change over time (co-researchers gradually became more confident in their skills). →show flexibility & adapt to the changes of circumstances. **Challenge:** Try and minimize the support workers’ input as much as possible.  **Good planning**: the venue (i.e., the research base) needs to be easily accessible for co-researchers, time of travel, scheduling team meetings well ahead of time, support to arrange travel or to organize for personal assistants to be present at research sessions; keeping the carers well-informed about how involvement is proceeding, may arise the need for psychological support, salary for co-researchers should be budgeted for in research planning  **Working with people with cognitive impairment:** most common strategies used were visual aids (e.g. colored arrows, laminated cards), put emphasis on being able to capture the non-verbal cues (may point to co-researcher feeling overwhelmed or stressed). | **Working with people with cognitive impairment**: memory problems, difficulties in expressive or receptive language or information processing | **Impact on Co-researcher & community**: personal development, feel empowered & in control, develop a more assertive attitude & a sense of pride, co-researchers becoming role models & advocates for their peers, skills developed during co-research can be transferred & used for future employment opportunities or in daily living, allowing participants to open up more easily about their experience, when participants had severe impairment & experienced difficulties in understanding the interview questions, the co-researchers could help them by reformulating difficult statements in a more appropriate language  **Impact on academic researcher & academic institutions:** change of expectations & assumptions on how to conduct research, change of attitude towards co-researcher, understanding that the process of learning through co-research was mutual, much can be learned from individuals with lived experience  **Impact on research:** may help to tailor the questions user-friendly*,* concrete, specific & relevant for participants*;* unique insight & ideas that may contribute to research outputs; dissemination of findings, as co-researchers can ensure that findings are reported in a concise, accessible, & audience-specific format. |
| Florence_2023 | 7 Studies reported using principles of Guba & Lincoln’s fourth-generation evaluation |  | Key obstacles were conceptions of mental health (e.g., the biomedical model and psychopathology); systemic issues (e.g., tutelage, violence, poverty, and lack of access to healthcare and basic rights); power asymmetries (e.g., primacy of academic and professional knowledge, infantilizing service users, and disenfranchisement in treatment); biomedical model’s reductionist views of mental health, violence, poverty, and social exclusion |  |
| Hawke_2023 |  | **In general:** building strong relationships, improving researcher attitudes toward engagement & equalizing power dynamic, avoiding research jargon, conducting debriefs, ensuring clear communication, taking the time to develop rapport to create a safe space, build strong relationships  **Need for training:** for co-researcher to enables to contribute to research, for academic staff to enable them to work with PWLE and prevent tokenistic engagement  **Types of training recommended:** one-on-one training, workshops, matching of PLWE with academic researchers, initial training & ongoing. | **Implementation gaps at the broader institutional level**: gaps in funding (more and more flexible), institutional support, and the understanding of engagement among research ethics board  **Implementation gaps in day‑to‑day practice**:  (1) Clear, early planning of PWLE engagement  (2) Building relationships and rapport  (3) Providing appropriate training and mentorship  (4) Increasing diversity  (5) Embedding PWLE engagement in leadership |  |
| Jakobsson_2023 |  | Acknowledge power imbalances & ensure genuine collaboration, empower service users to express their views in meetings with academic researchers, effective communication, providing materials in multiple formats, creating a safe and informal environment, creative & inclusive design atmosphere (using non-digital materials e.g. post-it notes & pens), effective communication, important to communicate how study data will be stored as this might be a particular concern for co-researcher with a history of psychosis. | Challenges with safeguarding, power imbalances, mental health stigma, and high turnover among mental health professionals.  Must pay extra consideration to recruitment strategies & study settings, aiming to mitigate barriers that may affect participants’ ability or willingness to partake in research (e.g. economic disadvantage, mistrust for example of the internet)  Power differentials (where researchers maintain control of the process: for example, unilaterally deciding what materials to share from a data analysis and co-design event)  Existing preconceptions of user involvement and mental health stigma (e.g. reported patients’ past experiences with health professionals in which complaints during co-produced research were interpreted as a relapse in their condition)  High turnover in mental health personnel can also impact the relationship between service users and health care professionals | **Impact on co-researcher & community:** created a sense of meaning & purpose, empowered marginalized individuals  **Impact on research:** greater insight by harnessing lived experience, created a secure & informal environment, which encouraged mutual engagement & resulted in more in-depth understanding & exploration, more meaningful & valid results |
| Kowe_2022 |  | **Training for participatory research:** in two publications, a training course for co-researchers was offered; in one paper, training was mentioned for the lead researcher; and in one paper, training was offered for all people involved. |  | **Impact on academic researcher & academic institutions:** widening the perspectives & knowledge of researchers  **Impact on research**: increased depth of data, General strength for research, adaptation of methodological approach, validation of research ideas, priority setting in research, practical orientation of research  **Negative Impact:** PPI can lead to a lower data quality, emotional burden for academic researcher, lacking reliability of the results, changes in planned research (e.g. additional time) |
| Miah_2019 | 1 Study: open-ended questions in a paper format 1 Study: online survey, semi structured interview, group interview 1 Study: semi structured interviews, focus groups |  | Prioritizing time to complete intervention sessions | Mostly anecdotal effects, only a few studies with formal evaluation reported on the effect of PPI, the quality of the evidence is low  **Impact on research:** identifying & prioritizing research questions, development of intervention manual & activity workbook (language clarity, ideas for materials used in intervention), helped to understand reasons for non-adherence*,* new insights & endorsed researchers’ interpretations of the findings |
| Ragavan_2018 | Variety of evaluation designs (half process evaluations, outcome measures) | Important is the trust building process, hours of conversation & engagement with DV advocates & survivors, research team worked closely with the community service agencies and stakeholders for several years, responsiveness to the ongoing and evolving needs and priorities of all stakeholders | Programmatic challenges: attendance barriers, programs being too short or unsustainable, and staff being overworked  Lack of trust, communication barriers,  Busy schedules, childcare, transportation costs, and financial barriers. | **Impact on research**: unspecific positive impact |
| Reyes_2023 |  |  |  |  |
| Schilling_2017 |  | **Communication:** different strategies such as ensuring accessibility of information, adapting information, securing knowledge through refreshments and summaries, use of meaningful and non-suggestive task, setting a pace that is appropriate for all participants, use of visualizations  **Location:** people with old-age-related conditions may have limited mobility →choose a venue which were familiar to participants, share a domicile with PPI participants with dementia (additional time to build relationship), use outdoor spaces, venue should be accessible, clearly structured and a quiet place, space for work and leisure activities  **Relationship:** for people with dementia, bonding can be difficult → relationships had to be renewed regularly, using relaxed environment or sharing a neutral space with PPI participants, spending both free and working time together for a few days  **Timing:** choose type & level of involvement to less time consuming methods, postal consultations were used instead of personal encounters, allowed participation on their own terms, set a schedule that included enough time for discussions & breaks, tailoring processes to the needs of the individuals, making attendance flexible, using different PPI methods interchangeably as needed  **Continuity:** way of recruitment as strategy to compensate for limitations in continuity:  **Support for Participants**: clarified roles at the beginning, trained reference group members on research methods, handed out a glossary of research terms, giving Participants sufficient time to share their impressions and feelings, offering additional support. | People with old-age-related conditions may have limited mobility, so the choice of the venue where involvement takes place is of great relevance; for people with dementia, bonding can be difficult; Hierarchies between researchers & participants;  **Diversity:** avoidance of tokenism was experienced as a challenge that needs to be addressed.  **Communication:** arose from poor memory, slow cognitive progressing, limited chronological reference, reduced confidence to make a contribution due to participants realizing that their abilities are vanishing and leading to a tendency to agree with researcher’s suggestions.  **Timing:** for researchers the timing of PPI is essential as involvement takes time (e.g., for building relationships). Further, potential participants may have temporal constraints or get tired more easily.  **Continuity:** the continuity of participation of people with old-age related conditions may be limited due to the progression of illness and related difficulties in care |  |
| Sheikan_2023 |  | **Co-Researcher level:** training and/or mentorship, feeling accepted and valued; having supports and resources available, fair compensation, trust; self‐awareness; seeing their feedback incorporated, contributions are formally recognized, continuity with roles, given time to contribute; having supports and resources available; included in consensus‐building or decision‐making; co‐chairing meetings; expertise in addition to lived experience ;.  **Academic researcher level:** recognizing power differences, valuing lived experience as expertise, engagement training and mentorship,  **Team level:** engaging PWLE at early stages in research process, flexibility throughout the research process, communication facilitators include holding pre- and de-briefs, transparent & clear communication, clearly defining roles, team interactions, supportive and respectful teams, inclusive, safe, and non-judgmental team environment  **Institutional level:** expectations set by institutions for high levels of engagement facilitated impactful engagement. | **Academic researcher level:** included researchers valuing institutional knowledge over lived experience and pushback from researchers,  **Lived experience level:** intrapersonal factors (feeling anxious or nervous; feeling isolated from other community partners; feeling disconnected, unsupported, or disengaged; skepticism, mistrust, or perceived risks; negative experiences; attendance issues; varying levels of interest and availability); Roles and responsibilities (having their other identities ignored; given limited information; not compensated); learning curve (Technological barriers; language barriers; travel and geographical barriers); PWLE feeling disconnected, unsupported, or disengaged, in addition to skepticism, mistrust, or perceived risks  **Team level**: lack of PWLE diversity among and limited engagement in early stages. Barriers at the team level include the use of jargon among team members. However, tokenism and conflicting views are frequent barriers to the successful engagement of PWLE.  **Institutional level**: power differences, the competitive nature of the research environment (e.g., fast‐paced, heavy focus on outputs), research culture itself, time constraints and limited funding were major barriers. | **Impact on Co-researcher & Community:** mutual learning, building knowledge & skills, personal & professional growth, PPI is a meaningful, empowering, & rewarding experience, feeling heard & valued, building confidence, feeling less alone, positive impact on their mental health & recovery, create a safe space for participants  **Impact on academic researcher & academic institutions:** fostered reciprocal learning between PWLE & researchers, enabled a positive change in the organizational culture, widening perspective, giving a deeper understanding  **Impact on research**: engagement positively influenced research quality, research components, & the research environment, improved decision‐making & communication, added value to their research, increased the retention of study participants, ensure that research was accessible, acceptable, & relevant to participants, appropriate study materials, perceived improvement of data & findings (data interpretation, analysis, reflexivity, authenticity & accuracy of findings, trustworthiness & credibility of data, depth & richness of data, validity), strengthened study design & methods (data collection for qualitative studies, improved interview guides), increase relevance to the community & service users, defining & refining research questions, improving the ethics review process by further highlighting ethical issues, increased recruitment, improve knowledge dissemination activities & service design |
| Souleymanov_2016 |  | Participants (especially those who are affiliated with community based agencies) should be offered employment, salaries, recognition, training, opportunities to participate in decision-making, offer opportunities to publish while sharing decision making process, importance of overcoming stereotypes, treating each person with dignity, praising accomplishments, developing a “trusted” presence in the drug-user community, frank discussions among collaborators, and acknowledging different agendas as a means to overcome communication barriers  Researchers recommended accommodating people in research at different times and in ways that shift in accordance with their health status, drug use patterns (and consequences), housing concerns, and other aspects of their lives | Possible test positive for illicit substance use, too intoxicated to participate in research  Significant barriers for marginalized people who use drug to complete training and gain employment: ways homelessness, as well as arrests for drug-related or other charges  Class-based and ableist biases implicit in some CBPR projects: e.g. all partners must demonstrate equitable participation by remaining actively involved in all phases of the research process. | **Impact on co-researcher & community:** empowerment, increased tolerance of drug using behaviours, reduction of stigma, & increased social support networks  **Impact on academic researcher & academic institutions**: increased tolerance of drug using behaviours  **Impact on research**: inclusion of culturally appropriate questions, sampling design that is reflective of the community needs |
| Stacciarini_2009 |  | Careful assessment of physical space and social environments (e.g., interpersonal relationships and extra workplace demands), substantial time to clarify their roles and to help them build and maintain positive relationships with co-workers | Identified in all publications: extra time needed to implement CBPR, methodological concerns  May overwhelming to coordinate activities involving all stakeholders in CBPR, including group meetings, project/research planning, and obtaining funds to support community and academic infrastructure.  Logistical challenge: scheduling meetings at convenient times for researchers and community members |  |
| Stacciarini_2010 |  |  |  | **Impact on research**: identifying suitable data collection procedures & in creating/adapting instruments that are culturally sensitive to the target community |
| Totzeck_2024 |  | **Preparation before commencement:** determine level of participation, plan demand-driven study design, clearly state aims, roles, & tasks to all members, define outcomes addressed by PPI members & document their input methods, utilize PPI guidelines & train the research team, skill based mentoring, prepare study information & informed consent in plain language, PPI facilitator to support moderation, methods & dissemination, plan sufficient time buffers, utilize youth friendly locations, use agendas for each meeting, calculate an adequate budget (including team building activities)  **Recruitment:** consider age-, gender, & cultural specificities  **Implementation (of PPI Groups):** avoid meetings on weekends & school holidays, create an informal environment as well as a safe space for open discussions, co-design/co-deliver trainings to co-researchers to reduce power restraints, fluid & flexible advisory group,  **Communication:** involve young people in determining the best methods of communication, flexibility in communication methods, communication also during funding gaps, team-building activities, end meetings by seeking feedback, address misunderstandings, & answering questions, evaluate meetings with VAS, Foster shared motivation, allow young members to lead meetings, make decisions through consensus & determine priorities, take observational notes to evaluate research development,  **Specificities Mental Health/Disorders:** involve multiple co-researchers for potential drop-outs, offer continuous support for psychological distress throughout the research process, consider the remission phase as a more suitable time for participation than the acute phase of any mental disorder  **Finalizing Study:** emphasize transparency in the research process to make young people feel respected & engaged, allow PPI group members to take ownership by participating in the dissemination of findings (conferences, podcasts, blog posts, plain language summaries), address the ethical issue of participant anonymity versus authorship | **Preparation & planning**: involving young people is time consuming, scheduling research meetings is difficult because of different obligations of young people, lack of sustainable funding for PPI projects, great financial & personal demand for PPI of young people  **Recruitment & Implementation:** difficulties in creating a heterogeneous, representative group in terms of gender and ethnicity, lack of young people in research team leads to lack of impact research, fluctuating memberships of young people lead to the necessity of ongoing recruitment  **Data Acquisition**: keeping in touch with young people over time can be challenging; lack of joint understanding, practice and knowledge of research procedures; disclosure research team membership might be sensitive; mental health related issues can influence involvement  **Finalizing Study & Evaluation:** involving young people in writing and disseminating professional reports is challenging; research teams consisting of professional and young researches are faced with different research priorities; young people as participants and authors have to consider if they want to disclosure personal life experiences | No quantitative data about their experiences in participation. |
| Valdez_2020 | Distal measures for evaluating YPAR (versus substance use) | Build trust between researchers and youth | Researchers were less likely to involve youth in development of the research question or in research design, youth and staff turnover, limited time to complete deliverables, limited resources and budget, challenges related to sustainability and achieving long-term impact | Mutual learning among youth & researchers. |

VAS = visual analogue scale, PWLD = People living with dementia, PWLE = People with lived experience, PPI Patient and Public involvement, CBPR = community-based participatory research, YPAR = Youth Participatory Action Research
